# Transfusion-induced HLA antibodies are short-lived and rarely recur post-heart transplantation: a single-center retrospective study with implications for virtual crossmatching

**DOI:** 10.3389/fimmu.2026.1729124

**Published:** 2026-01-21

**Authors:** Raja Rajalingam, Stalinraja Maruthamuthu, Brian R. Shy, Julia Cunniffe, David Lowe, Othman A. Aljohani, Georg Wieselthaler

**Affiliations:** 1 Immunogenetics and Transplantation Laboratory, Department of Surgery, University of California, San Francisco, San Francisco, CA, United States; 2 Department of Laboratory Medicine, University of California, San Francisco, San Francisco, CA, United States; 3 Heart Transplant and Mechanical Circulatory Support Program, Department of Surgery, University of California, San Francisco, San Francisco, CA, United States; 4 Division of Pediatric Cardiology, Department of Pediatrics, University of California, San Francisco, San Francisco, CA, United States

**Keywords:** alloimmunization, blood transfusion, heart transplantation, HLA antibodies, sensitization, transfusion-induced antibodies, ventricular assist device

## Abstract

**Introduction:**

The development of human leukocyte antigen (HLA) antibodies poses a major challenge in transplantation by limiting donor compatibility and increasing the risk of graft failure. In patients with advanced heart failure, ventricular assist devices (VADs) are frequently used as a bridge to transplantation and often necessitate perioperative blood transfusions, a recognized trigger for HLA sensitization. However, the risk factors, timing, and clinical relevance of transfusion-induced HLA antibodies in VAD recipients remain incompletely defined.

**Methods:**

We conducted a retrospective analysis of 60 adult heart transplant candidates who underwent VAD implantation and received blood transfusions. HLA antibody profiles were assessed at three time points: pre-VAD implantation, post-VAD/pre-transplantation, and post-transplantation. Patients were categorized according to the number of newly detected HLA antibodies following VAD implantation as non-producers (NP; no new antibodies), low producers (LP; 1–10 antibodies), or high producers (HP; >10 antibodies). Associations between antibody development and demographic characteristics, transfusion exposure, pre-existing sensitization, antibody persistence, and transplant outcomes were evaluated.

**Results:**

Following VAD implantation, 73% of patients developed new HLA antibodies, with 65% of these antibodies emerging during the post-VAD/pre-transplant interval. Patient distribution was 35.0% NP, 51.7% LP, and 13.3% HP. Age, ethnicity, and transplant rates were comparable across groups; however, females were disproportionately represented in the HP group (75%, 6 of 8). Neither the number nor the type of blood products transfused was associated with antibody development. The majority of transfusion-associated HLA antibodies were transient, declining rapidly and rarely recurring after transplantation. In contrast, persistent antibodies were predominantly pre-existing and were more frequently observed in female patients, consistent with prior sensitization events such as pregnancy. Notably, unsensitized male patients demonstrated minimal antibody formation following transfusion.

**Discussion:**

These findings indicate that transfusion-induced HLA antibodies in VAD recipients are generally transient and lack durable serologic significance, with little evidence of post-transplant rebound. In contrast, sustained alloimmune responses are primarily driven by pre-existing sensitization, particularly pregnancy-related exposure in female patients. These results support a more individualized approach to HLA antibody surveillance and virtual crossmatching, especially in patients with limited transfusion exposure and no prior sensitization.

## 1 Introduction

The development of human leukocyte antigen (HLA) antibodies remains a major obstacle to successful transplantation. Sensitization arises through exposure to non-self HLA allotypes, most commonly via organ transplantation, pregnancy, or blood transfusion (BT). In patients with advanced heart failure, ventricular assist devices (VADs) are often used as a bridge to heart transplantation (HTx). This therapy frequently requires perioperative transfusions, a well-recognized source of HLA sensitization in this population (1–4). The presence of HLA antibodies complicates transplantation by narrowing the pool of compatible donors, extending wait times, and, in some cases, necessitating HTx in the setting of preformed donor-specific antibodies (DSA)—a circumstance associated with increased post-transplant mortality, graft injury, dysfunction, and failure (5–9).

Despite this recognized risk, the durability, recall potential, and clinical consequences of transfusion-induced HLA antibodies remain poorly defined, particularly in transplant candidates. This knowledge gap has important implications for organ allocation policies, crossmatching strategies, and long-term outcomes after transplantation. Given the growing use of VADs across both adult and pediatric populations, and their pivotal role in managing advanced heart failure, it is critical to understand the immunologic effects of transfusion in this setting. In particular, it is unclear whether transfusion volume and timing alone drive antibody formation or whether pre-existing sensitization predisposes certain individuals to heightened alloimmune responses.

To address these questions, we conducted a retrospective analysis of 60 adult heart transplant candidates who underwent VAD implantation with transfusion support. Our study aimed to characterize the nature, persistence, and potential rebound of transfusion-induced HLA antibodies, including DSAs, in this cohort. Our findings promise to deepen the understanding of the immunologic consequences of blood transfusion in the pre-transplant setting, with broader implications for the management of sensitization in all transplant candidates who have been exposed to transfusions.

## 2 Materials and methods

### 2.1 Study population

This retrospective, single-center study included 60 adult patients who were bridged to heart transplantation with a VAD implantation at the University of California, San Francisco, between 2012 and 2017. Only primary durable left ventricular assist device (LVAD) implants were included—specifically, the HeartMate II (Thoratec, Pleasanton, California, USA) and the HeartWare Ventricular Assist System (HVAS) (HeartWare, Framingham, Massachusetts, USA). Patients with VAD re-implantation or a history of prior heart transplantation were excluded. Demographic and clinical data were extracted from electronic medical records. Information on both the number of units and the volume of packed red blood cells (pRBCs), plasma, and platelet transfusions was also collected (**Table 1**). All transfusions at UCSF are leukoreduced, CMV-negative, ABO-compatible, and irradiated in accordance with institutional protocol. HLA-matched platelets were not administered. All heart transplants were performed using a virtual crossmatch approach, as physical crossmatch results were not available at the time of transplantation. Retrospective flow cytometry crossmatches performed after transplantation were negative in all cases. The study was approved by the Institutional Review Board of the University of California, San Francisco (IRB #16-19103).

**Table 1:** Cohort characteristics.

	All (60)	Post-VAD antibody production			p-value
		None (21)	Low (31)	High (8)	
Age at VAD implant^a^	53.6 (24.4-71.1)	52.0 (28.8-71.1)	55.9 (24.4-68.6)	48.8 (24.9-63.5)	n.s.
Sex^b^					
Male	48 (80)	19 (90)	27 (87)	2 (25)	0.001552^c^
Female	12 (20)	2 (10)	4 (13)	**6 (75)**	
Race/Ethnicity^b^					
White	29 (48)	10 (48)	14 (45)	5 (62.5)	n.s.
Black	10 (17)	4 (19)	4 (13)	2 (25)	
Asian	8 (13)	2 (10)	6 (19)	0 (0)	
Hispanic/LatinX	9 (15)	2 (10)	6 (19)	1 (12.5)	
Other/Unknown	4 (7)	3 (14)	1 (3)	0 (0)	
Blood Products^a^					
Total	8.9 (0-50)	9.2 (0-26)	9.1 (0-50)	7.0 (0-20)	n.s.
pRBC	3.2 (0-22)	2.7 (0-12)	3.5 (0-22)	3.6 (0-10)	n.s.
Platelets	2.5 (0-12)	2.4 (0-4)	2.5 (0-12)	2.5 (0-10)	n.s.
Plasma	3.3 (0-20)	4.0 (0-14)	3.4 (0-20)	0.9 (0-2)	n.s.
Transplantation^b^					
Performed	47 (78)	18 (86)	25(81)	4 (50)	n.s.
With followup	41 (68)	16 (76)	22 (71)	3 (37.5)	
Number of HLA Antibodies^a^					
Pre-VAD	3.4 (0-62)	2.0 (0-8)	1.6 (0-6)	13.6 (0-62)	0.009^d^
New Post-VAD	7.7 (0-78)	0 (0-0)	3.3 (1-9)	45.3 (13-78)	n.t.^e^
Total Post-VAD	10.4 (0-88)	0.9(0-4)	4.7 (1-11)	57.3 (13-88)	n.t.^e^
New Post-Transplant	2.3 (0-28)	0.8 (0-6)	3.4 (0-28)	3.3 (1-8)	n.s.
Total Post-Transplant	5.1 (0-37)	1.5 (0-6)	6.0 (0-32)	19.7 (10-37)	0.00024^d^
Total	12.7 (0-100)	2.7 (0-9)	7.3 (1-35)	60.1 (21-100)	n.t.^e^

a = Mean(Min-Max).

b = N(%).

c = chi-square.

d = ANOVA.

e includes variable used for categorization; not tested.

### 2.2 Immunosuppression and desensitization management

No patients received desensitization therapy (such as plasmapheresis, IVIG, rituximab, bortezomib, or photopheresis) during the VAD implantation period. Furthermore, none of these therapies were administered prior to heart transplant listing or transplantation. Standard post–heart transplant immunosuppression typically includes induction therapy with anti-thymocyte globulin, followed by maintenance triple therapy consisting of a calcineurin inhibitor, mycophenolate mofetil, and prednisone. Corticosteroids are gradually tapered, and mTOR inhibitors may be substituted for calcineurin inhibitors in patients with renal dysfunction or cardiac allograft vasculopathy. Immunosuppressive regimens are tailored to individual needs, based on the risk of rejection, infection, and renal function.

### 2.3 HLA antibody screening and analysis

The serum samples were pretreated with dithiothreitol (DTT) to minimize assay interference and to prevent the prozone effect, a phenomenon in which high-titer antibodies aggregate on antigen-coated beads, leading to false-negative results. HLA antibody testing was performed using Luminex-based Single Antigen Bead (SAB) assays (LABScreen SAB, Thermo Fisher Scientific, Carlsbad, CA) according to the manufacturer’s instructions. To improve reproducibility and reduce inter-assay variability, an automated liquid-handling system (LABXpress Pipettor, Thermo Fisher Scientific) was used for sample processing. All reactions were run on the Luminex FlexMap 3D platform (Luminex Corporation, Austin, TX). To exclude self-reactive antibodies, patients underwent HLA typing (HLA-A, -B, -C, -DRB1, -DRB3, -DRB4, -DRB5, -DQA1, -DQB1, -DPA1, and -DPB1) using sequence-specific oligonucleotide (SSO) hybridization with the LabType^®^ SSO kit (One Lambda, Canoga Park, CA) as previously described by dela Cruz et al (10).

Fluorescence signals were reported as mean fluorescence intensity (MFI), normalized using the manufacturer-recommended divider of 1.67. Data were analyzed with HLA Fusion software (Thermo Fisher Scientific). Antibody measurements were performed using defined bead lots and standardized procedures to ensure reproducibility across study years. The following bead lots were used: 2012–2014, SA1_LOT-7 and SA2_LOT-9; 2015–2016, SA1_LOT-9 and SA2_LOT-10; and 2017, SA1_LOT-10 and SA2_LOT-12. Inter-lot verification was conducted by comparing mean fluorescence intensity (MFI) values obtained with older and newer bead lots using five standardized positive and two negative laboratory serum controls. No systematic bias was observed between lots, and therefore no normalization adjustments were required. Background signal was assessed using blank beads and antibody-free control serum and buffer, and normalized MFI values were corrected for background fluorescence. Coefficients of variation (CVs) for key internal controls demonstrated high reproducibility, with intra-assay CVs ranging from 4.3–7.2% and inter-assay CVs from 6.8–9.5%, consistent with established reproducibility standards for multiplex immunoassays.

Antibody assignment was determined by identifying cross-reactivity patterns among beads sharing public epitopes, with reactivity defined as a normalized MFI ≥1000. When antibody specificity included multiple allelic variants (e.g., HLA-A*02:01, *02:03, *02:06 within the HLA-A2 group) and all variants exceeded the positivity threshold, the average MFI was calculated to represent the overall antibody strength. If only a subset of variants was reactive, each positive bead was reported individually with its corresponding MFI. New HLA antibodies were defined as those that emerged after VAD implantation but were absent beforehand.

### 2.4 Study groups and statistical analysis

The presence of HLA antibodies was evaluated at three time points: prior to VAD implantation, after VAD implantation but before transplantation, and following heart transplantation. Patients were classified according to the number of new antibodies detected during the post-VAD, pre-transplant interval. Three groups were defined: high producers (HP; >10 antibodies), low producers (LP; 1–10 antibodies), and non-producers (NP; no new antibodies detected).

All statistical analyses were conducted in R (11). Categorical variables (sex and ethnicity) were compared using Pearson’s chi-square test. Differences in the number of blood products administered were visualized in R, with significance tested by ANOVA followed by pairwise t-tests with Bonferroni correction. A two-sided p-value< 0.05 was considered statistically significant.

## 3 Results

### 3.1 Sex-specific risk of HLA sensitization following transfusion in VAD recipients

**Figure 1:**
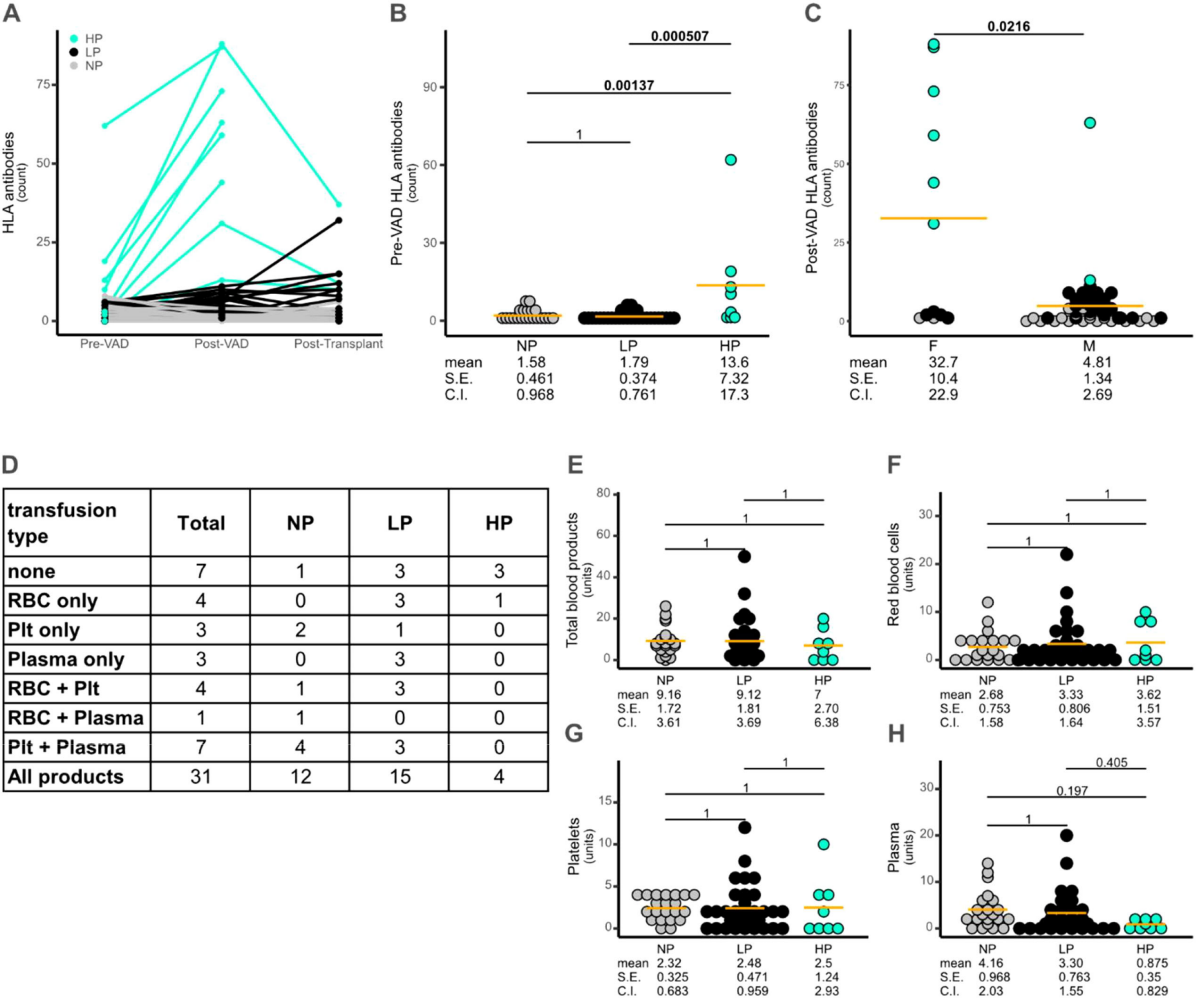
HLA antibody production correlates with the presence of pre-existing antibodies, rather than with the type or frequency of transfusion. **(A)** Cohort members were categorized by the number of new HLA antibodies arising in the post-VAD period. **(B)** Shows a significant difference in the number of pre-existing HLA antibodies in the HP group. Pairwise t-test values are shown in the figure. Anova, *F*(1,58) = 7.25, *p* = 0.009, $\eta_{g}^{2}$ = 0.11. **(C)** Shows a significant difference in the number of novel post-VAD antibodies produced by females versus males. The pairwise t-test value is shown in the figure. **(D)** Shows the types of transfusions performed with the number of individuals receiving each combination. **(E-H)** Show correlation between antibody production category and type and number of blood transfusion units. No statistical significance was found. Pairwise t-test values are shown in the figures. Anova values were **(D)***F*(1,58) = 0.22, *p* = 0.64, $\eta_{g}^{2}$ = 0.004) **(E)***F*(1,58) = 0.38, *p* = 0.54, $\eta_{g}^{2}$ = 0.007 F) *F*(1,58) = 0.002, *p* = 0.96, $\eta_{g}^{2}$ = 4.3e−05 and G) *F*(1,58) = 2.97, *p* = 0.09, $\eta_{g}^{2}$ = 0.05. NP - No producers (no new antibodies); LP - low producers (1–10 new antibodies); HP - high producers (>10 new antibodies).

We conducted a retrospective analysis of 60 patients who underwent VAD implantation as a bridge to heart transplantation. Based on the number of new HLA antibodies detected between VAD implantation and transplantation, 35.0% were classified as non-producers (NP; no new antibodies), 51.7% as low producers (LP; 1–10 antibodies), and 13.3% as high producers (HP; >10 antibodies) (**Figure 1A**, **Table 1**). No significant differences were observed in age, ethnicity, or likelihood of proceeding to transplantation among the groups (**Table 1**). However, the HP cohort had a higher number of pre-existing HLA antibodies (**Figure 1B**), and females were disproportionately represented in the HP group, comprising 75% (6 of 8) of that subgroup (**Figure 1C**, **Table 1**). To further investigate this finding, we reviewed the available medical records and extracted parity and gravidity data for all 12 female patients. The mean number of pregnancies was 2.1. Among these, six of the twelve females with pre-existing HLA antibodies who were also classified as high antibody producers following VAD implantation had experienced at least two pregnancies. However, paternal HLA typing data were unavailable, limiting our ability to assess potential HLA mismatch exposures that may have contributed to the development of pre-existing HLA antibodies.

### 3.2 Transfusion characteristics do not correlate with HLA antibody production

We assessed whether the number and type of blood products administered correlated with the development of post-VAD HLA antibodies. No significant differences were observed among the three groups (**Table 1**, **Figures 1D–G**).

### 3.3 Overall antibody responses across study phases

Antibody responses were evaluated at regular intervals during the post-VAD and post-transplant periods. Pre-existing HLA antibodies were identified in 38 of 60 patients (63%), comprising 29 males (48%) and 9 females (50%) (**Table 2**). Across all study phases (pre-VAD, post-VAD, and post-transplant), 54 of 60 patients (90%) demonstrated HLA antibody production, while 6 males (10%) showed no antibody formation (**Table 2**). Newly formed HLA antibodies were detected in 73% of the cohort (34 males [71%] and 10 females [83%]). Most new antibody responses occurred during the post-VAD interval (39 patients, 65%), whereas fewer were observed in the post-transplant interval (21 patients, 35%) (**Table 2**).

**Table 2:** Individuals with HLA antibodies.

	Post-VAD antibody production			
	All (60)	None (21)	Low (31)	High (8)
	48 M	19 M	27 M	2 M
	12 F	2 F	4 F	6 F
Pre-existing HLA antibodies				
Male	29	11	17	1
Female	9	2	2	5
New post-VAD HLA antibodies				
Male	29	0	27	2
Female	10	0	4	6
Post-VAD HLA antibodies				
Male	38	9	27	2
Female	12	2	4	6
New post-transplant HLA antibodies				
Male	18	5	12	1
Female	3	0	1	2
Post-transplant HLA antibodies				
Male	25	8	16	1
Female	5	1	2	2
No HLA antibodies				
Male	6	6	0	0
Female	0	0	0	0
No new HLA antibodies				
Male	14	14	0	0
Female	2	2	0	0
New HLA antibodies				
Male	34	5	27	2
Female	10	0	4	6
All HLA antibodies				
Male	42	13	27	2
Female	12	2	4	6

Patients in the HP group exhibited significantly higher numbers of pre-existing HLA antibodies prior to VAD implantation (**Figure 1B**, **Table 1**). When only newly formed antibodies were assessed in the post-transplant period, no significant differences were observed among groups (**Table 1**). In contrast, when both pre-existing and new antibodies were included, a significant difference persisted, driven by elevated baseline sensitization in the HP group. Pairwise t-tests confirmed that this effect was attributable to differences between HP and the other groups, with no significant differences between NP and LP.

### 3.4 Antibody kinetics and patient subgroup analysis

**Figure 2:**
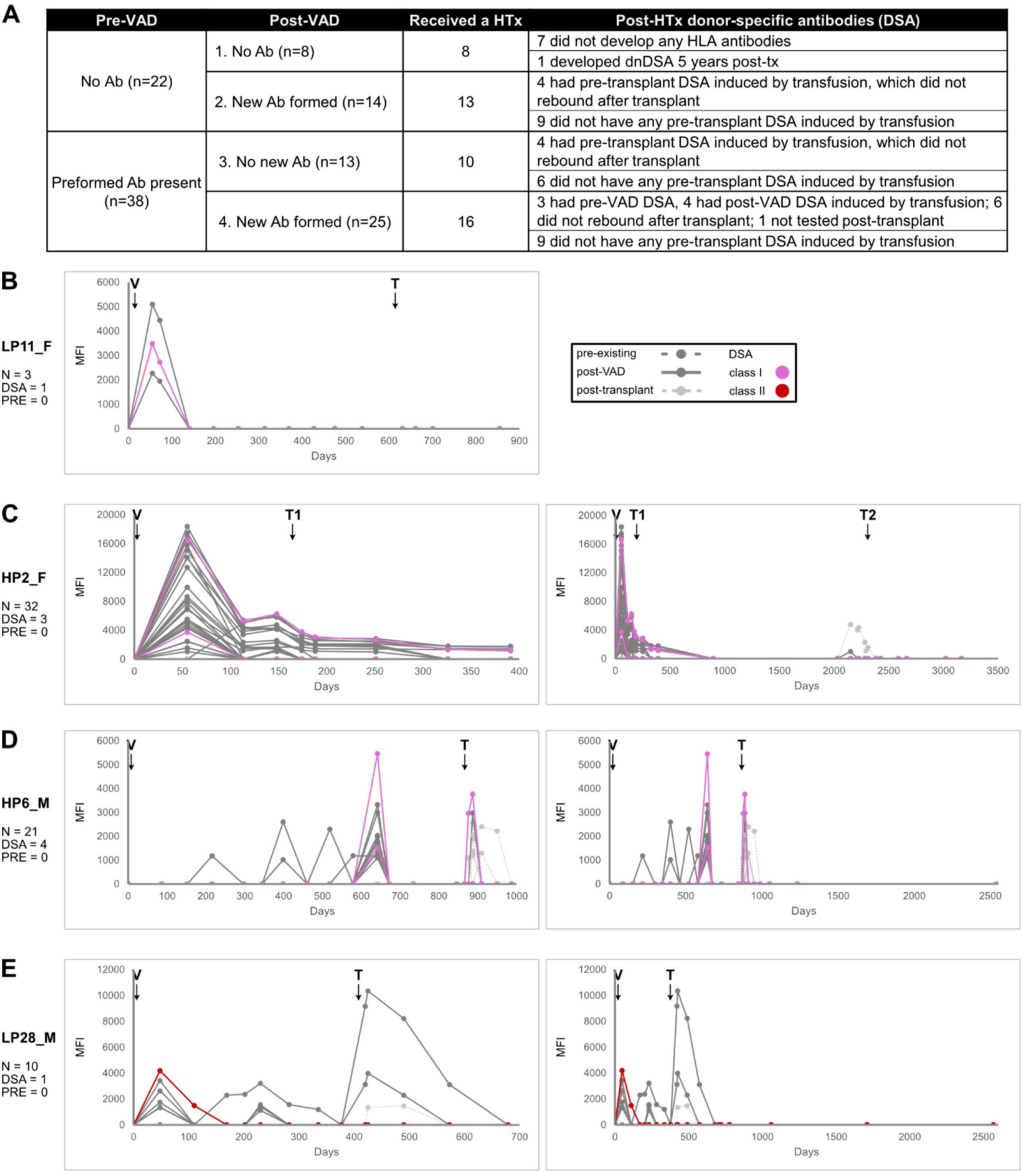
HLA antibodies rise and fall rapidly. **(A)** shows the 4 groups that cohort members were assigned based on the acquisition of new antibodies and the existence of pre-existing antibodies. Also noted is whether a transplant occurred. **(B-D)** Antibody trajectory plot of a group 2 members. V indicates VAD implantation, T indicates transplantation, _F after patient identifier indicates female, and _M indicates male. For **(D, E)**, an expanded view of the peri-VAD interval is shown in the left panel; the complete trajectory is on the right. The patient in C received two transplants, shown as T1 and T2. The legend for all trajectory plots is shown to the right in part **(B)** Pre-existing antibodies are shown with a dashed line. New antibodies are shown with solid lines, dark gray for those arising post-VAD and light gray for those arising post-transplant. DSA are colored according to the key shown.

In addition to the six patients without HLA antibodies, ten patients (17%) demonstrated only pre-existing antibodies. Based on antibody acquisition patterns, pre-existing status, and transplant outcome, patients were stratified into 4 groups (**Figure 2A**). Groups 1 and 2 had no pre-existing antibodies. Of these 22 patients, 8 (36%, group 1) formed no new antibodies post-VAD implantation, while the remaining 14 (64%, group 2) produced new antibodies. Pre-existing antibodies were present in the 38 patients of groups 3 and 4, with 13 (34%, group 3) forming no new antibodies and 25 (66%, group 4) producing new antibodies.

All patients in group 1 received a heart transplant, with seven out of eight having no development of HLA antibodies post-transplant. The exception was the patient developing a *de novo* DSA 5 years post-transplant. Thirteen of fourteen (93%) of the group 2 patients received a transplant. Four of these patients had transfusion-induced DSA prior to transplant that did not rebound afterward. **Figure 2B** shows one of these patients. This patient developed three new HLA antibodies post-VAD implantation, including a donor-specific class I antibody. These responses were short-lived and did not recur after transplantation (**Figure 2B**; the complete set of antibody trajectories is shown in **Supplementary Figure 1**).

The remaining group 2 patients developed transient antibodies post-transplant (**Figures 2C-E**). All three had at least one new HLA antibody form post-transplant. In the patient shown in **Figure 2C** (left panel), two class I donor-specific antibodies were detected, one of which corresponded to a second transplantation. The right panel illustrates the complete antibody trajectory, highlighting the appearance of a *de novo* antibody immediately before the second transplant, while both class I antibodies failed to recur. The patient shown in **Figure 2D** demonstrated multiple antibody spikes in the post-VAD interval, with the final spiking including two class I DSAs. Post-transplant, one of these antibodies recurred together with additional antibodies; however, the response was again transient. **Figure 2E** shows the final group of 2 patients with antibody development post-transplant. This patient had a single class II transfusion-induced antibody that did not recur post-transplant.

**Figure 3:**
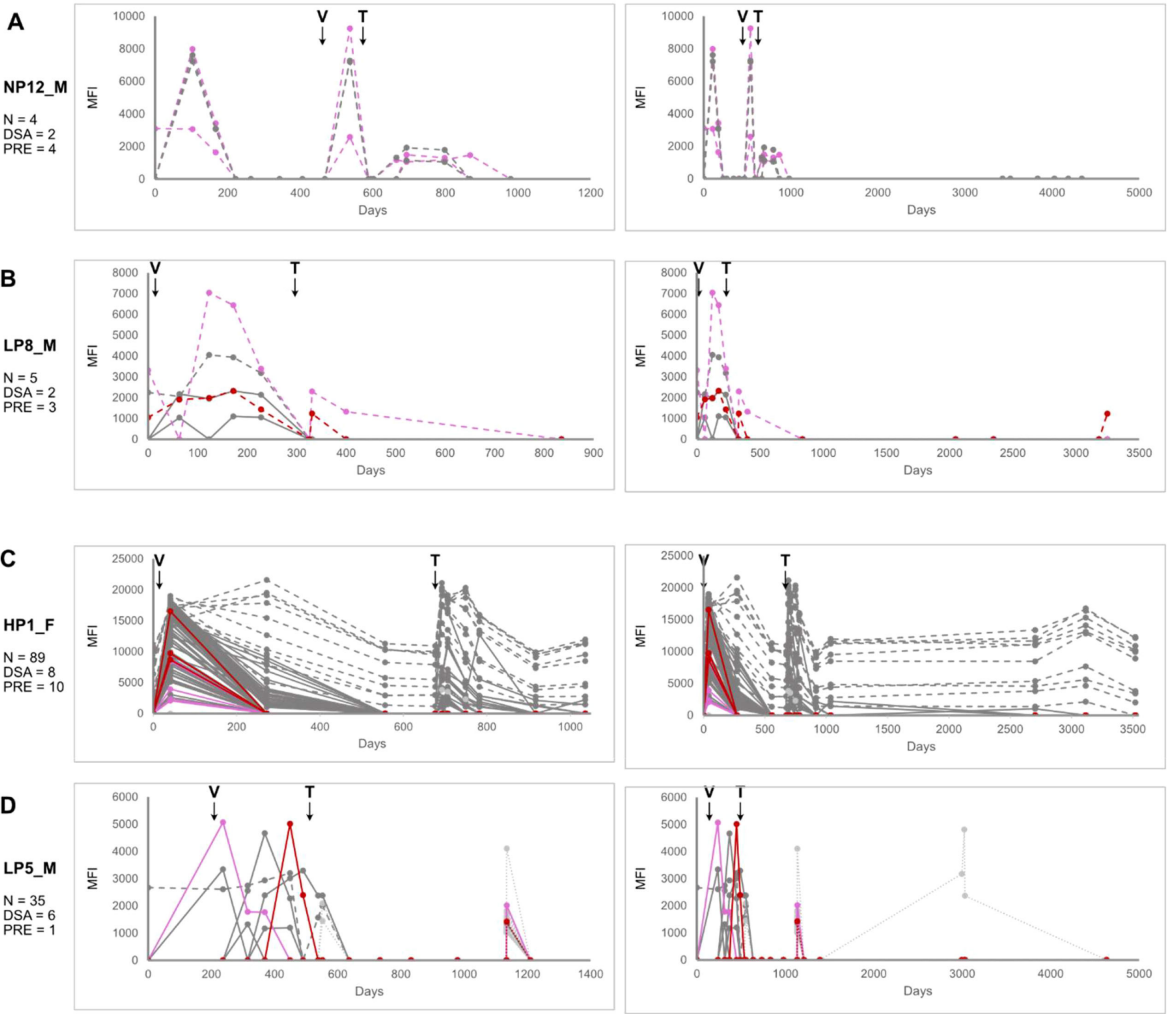
Some pre-existing HLA antibodies may persist for a long time. Example antibody trajectories of **(A)** group 3, with only pre-existing HLA antibodies, and **(B-D)** group 4. Line types and colors are the same as in **Figure 2**.

Transplantation rates were lower for the patients with pre-existing antibodies, with ten (77%) group 3 and 16 (64%) group 4 patients receiving transplants. Similar patterns of new antibody acquisition and kinetics were observed in patients with pre-existing antibodies compared to those without (examples shown in **Figure 3**; all trajectories are presented in **Supplementary Figure 1**).

The patient in **Figure 3A** (group 3) had only pre-existing antibodies, likely due to prior therapeutic interventions. Two were class I DSAs, which showed transient spikes before and after VAD implantation and immediately post-transplant, but were absent thereafter. Group 4 patients, who also had pre-existing antibodies, exhibited new antibody formation following VAD implantation (**Figures 3B-D**). **Figure 3B** shows a patient with pre-existing rose during the post-VAD interval, along with the acquisition of new antibodies. There was then a small, transient peak of antibody immediately post-transplant (particularly class I DSAs), but they subsequently declined, resembling the pattern exhibited by the patient shown in **Figure 2D**.

Not all pre-existing antibodies disappeared over time. For example, the patient in **Figure 3C** had pre-existing non-DSAs that persisted throughout the entire observation period of nearly 10 years. This patient, a female, likely developed these antibodies during pregnancy. Additional group 4 members exhibited antibody kinetics resembling those of the patient shown in **Figure 2D**, with multiple transient spikes (**Figure 3D**). In the case shown, one pre-existing antibody was not sustained, and two post-transplant spikes occurred. The first spike included a pre-existing class II DSA along with two additional class I DSAs and 23 non-DSAs.

### 3.5 Technical considerations in antibody kinetics

Interpretation of antibody trajectories was influenced by assay timing. As illustrated in the right panel of **Figure 3D**, an antibody appearing around day 3000 was represented as a broad peak, an artifact of infrequent sampling. Closer inspection revealed a rapid rise and fall over three consecutive assays, suggesting the broad appearance was an overestimation. Thus, peak widths should be interpreted as maximal approximations, with the understanding that many antibody responses were in fact narrower and more transient than depicted.

### 3.6 Acquisition and persistence of antibody responses

**Figure 4:**
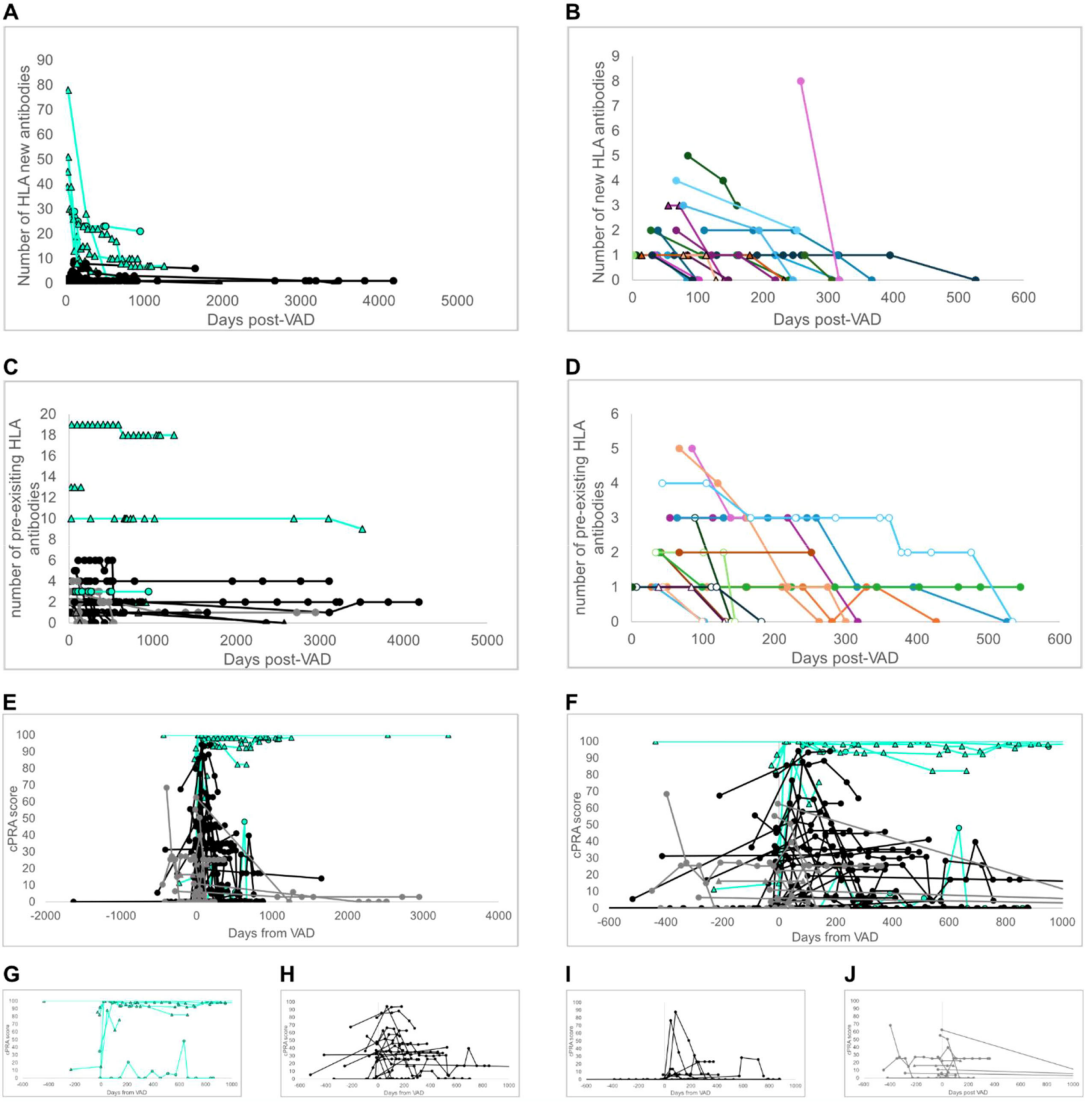
Post-VAD implantation antibodies are short-lived. Determining the persistence of HLA antibodies post-VAD is constrained by the timing of HLA antibody assays. **(A)** shows the number of new antibodies present post-VAD implantation. Curves end at the final assay or the final assay pre-transplant. HP individuals are shown in aqua, and LP individuals are shown in black. Females are represented by triangles and males by circles. Only individuals with at least two post-VAD assays and the first occurring within one year of VAD implantation are shown. **(B)** This is the same data as in **(A)** showing individuals with fewer than 9 new antibodies (all are LP) and for which there was no antibody persistence beyond d600 post-VAD. **(C)** shows the persistence of pre-existing antibodies. Color coding is the same as panel A, with the addition of dark gray for NP individuals. **(D)** shows the same data without individuals having antibody persistence beyond d600 post-VAD. Open markers indicate NP individuals; all others are LP. **(E)** shows cPRA scores with d0 marking VAD implantation. Color coding is the same as for **(C, F)** is the same data as **(E)**, limited to 600 days pre/post VAD. **(G-J)** is the data from panel **(F)** divided into HP **(G)**, LP with pre-existing antibodies **(H)**, LP with no pre-existing antibodies **(I)**, and NP with pre-existing antibodies **(J)**.

We determined the persistence of HLA antibodies acquired at the time of VAD implantation by restricting our analysis to patients who were positive for at least one new HLA antibody at the first post-VAD assay point, which occurred within one year of VAD implantation. This resulted in a cohort of 31 patients. The number of new antibodies formed and their persistence are shown in **Figure 4**. Panel A illustrates the rapid decline of most antibodies. There were 338 new post-VAD antibodies in this cohort, of which 40 (12%) remained persistent in the past two years. In contrast there was a decline of 21% (267 antibodies) by day 100 post-VAD, 41% (201 antibodies) by day 180, 68% (107 antibodies) by one year and 83% (59 antibodies) by two years post-VAD The persistent antibodies came from five individuals, two LP individuals with one antibody each and three HP individuals with 21, 10 and 7 antibodies. A zoomed-in look at the antibody decline in the first two years is shown in panel B.

We also assessed the persistence of pre-existing HLA antibodies. **Figure 4C** shows the trajectories of pre-existing antibodies. They fall into two groups: those that remain persistent and those that exhibit a decline similar to the newly formed post-VAD antibodies, as shown in the zoomed-in depiction in **Figure 4D**. Of the initial 105 pre-existing antibodies, 61 remained present after 2 years. There were in 13 individuals with a range of 1–18 antibodies present. Although the overall proportion of patients having pre-existing HLA antibodies was similar between males and females (60.4% vs 67.7%) the burden of antibodies differed. Female patients ranged from 0 to 62 pre-existing antibodies, with an average of 9.1 pre-existing antibodies, 4.9 class I, and 4.2 class II. This compared to the male patients, who ranged from 0–8 pre-existing antibodies and averaged only 1.9 pre-existing antibodies, 1.4 class I, and 0.5 class II.

### 3.7 Effect of antibody acquisition and persistence on calculated panel reactive antibody values

We calculated the cPRA scores for all individuals (**Figures 4E-J**). The values are displayed with d0 representing the day of VAD implantation. The plot is truncated at the last assay pre-transplant. As expected, the acquisition of new antibodies followed by a rapid decline introduces sharp spikes in the cPRA scores for some individuals. There is a lesser effect on those with high levels of pre-existing antibodies (**Figure 4G**) where the cPRA score begins and is maintained at a high level. In other cases, the acquisition and decline of new post-VAD antibodies result in transient spikes in cPRA scores (**Figures 4H, I**), regardless of the presence or absence of pre-existing antibodies.

## 4 Discussion

In this study, blood transfusions administered during VAD implantation did not significantly induce HLA antibody formation in unsensitized male patients. In contrast, two-thirds of female patients had detectable HLA antibodies prior to implantation, and over 80% developed new antibodies afterward, consistent with a recall or memory response rather than true *de novo* sensitization. Our findings confirm that pre-existing HLA antibodies are a major risk factor for additional antibody development following VAD implantation, consistent with previous reports (1, 2). This pattern aligns with pregnancy-related sensitization, where exposure to fetal paternally inherited HLA antigens generates long-lasting alloimmune memory that can re-emerge with subsequent antigen exposure, such as from transfusions or inflammatory stimuli, including major surgery. These observations underscore the potential benefit of providing HLA-matched blood products to female patients at risk of sensitization.

The number and type of blood products administered perioperatively did not correlate with post-VAD HLA antibody formation, suggesting that transfusion exposure alone is not the primary determinant of sensitization in this cohort. All transfusions at UCSF are leukoreduced, CMV-negative, ABO-compatible, and irradiated in accordance with institutional protocol. These standardized transfusion practices may help explain the lack of association between the type of transfusion and antibody development. Our findings are consistent with prior studies (12–15) and likely reflect the widespread use of leukoreduced blood products, which reduces exposure to non-self HLA antigens. However, transfusion practices vary across centers, and the grouping of different blood components, combined with the relatively small number of patients receiving plasma or platelets, may obscure the individual contributions of these products.

Across all study phases, 90% of patients developed HLA antibodies, while 10% remained antibody-free. Newly formed antibodies were detected in nearly three-quarters of patients, most commonly after VAD implantation. The typical pattern of transfusion-induced antibodies was transient, characterized by a rapid rise followed by a decline, with little evidence of recurrence after transplantation, even up to four years post-transplantation. These observations suggest that transfusion-induced sensitization has limited lasting serologic impact and may be safely disregarded once antibody levels diminish on serial testing. While this study did not evaluate post-transplant clinical outcomes such as rejection or graft function, our findings indicate that transient antibody responses are primarily of immunologic rather than clinical relevance. Thus, our interpretations are restricted to serologic observations and their potential implications for virtual crossmatching and donor selection. These findings are consistent with prior large cohort studies, which reported no significant differences in rejection rates or mortality between sensitized and non-sensitized patients bridged to transplantation with VAD support (7). By contrast, persistent antibodies were more often pre-existing and occurred predominantly in female patients, further supporting the role of pregnancy-associated sensitization.

Among solid organ transplant waitlist candidates, blood transfusion is a well-recognized risk factor for alloimmunization (16). However, our findings suggest that limited transfusion exposure—defined as fewer than five units of red blood cells, five units of plasma, or three units of platelets—does not consistently lead to the formation of HLA antibodies. These observations carry important clinical implications. For waitlisted patients, routine antibody testing after minimal transfusion exposure may not always be warranted. When transfusions do not induce new HLA antibodies, virtual crossmatch results remain stable, thereby preserving donor compatibility, calculated panel reactive antibody (cPRA) levels, and predicted crossmatch outcomes. In such circumstances, reducing unnecessary antibody testing at the time of transplantation could help lower costs, prevent procedural delays, and shorten cold ischemia times, all while maintaining patient safety. Routine HLA antibody screening should still be performed for waitlisted candidates who have had large transfusions or prior sensitizing events, to maintain transplant safety and ensure the best donor matches.

Finally, while some centers pursue intraoperative desensitization strategies to reduce antibody burden and expand transplant access, our findings indicate that transfusion-induced antibodies are generally short-lived. A waiting period of approximately six months may allow for the natural decay of antibodies without the need for additional intervention. Notably, we found no evidence of antibody rebound after transplantation in patients who had transient, transfusion-induced sensitization.

This study has several limitations. As a single-center analysis with a modest sample size, statistical power may have been insufficient to detect all relevant risk factors. Furthermore, detailed histories of prior sensitizing events were not consistently available, and reliance on chart review for demographic and clinical data (including parity/gravidity history) may have introduced bias. Antibody testing was also performed as clinically indicated, rather than at standardized intervals, which limited the assessment of antibody kinetics.

In summary, our findings suggest that pre-existing sensitization—particularly among female patients—may be the predominant driver of HLA antibody development following VAD implantation, whereas transfusion burden alone does not appear to reliably predict sensitization. Transfusion-induced antibodies are generally transient and primarily influence serologic profiles rather than established clinical outcomes, whereas pregnancy-associated antibodies tend to be more persistent and clinically significant. These observations should be considered hypothesis-generating, and larger multicenter studies will be essential to validate whether these serologic trends translate into differences in transplant success or rejection rates.

## Data Availability

The raw data supporting the conclusions of this article will be made available by the authors, without undue reservation.

## Supplementary material

Supplementary Figure 1 Antibody profiles are shown for all cohort members, divided into four major groups outlined in **Figure 2**. For the group 1 patients who had no preformed antibodies and no new pre-transplant antibodies, the timing of the assays for each patient is shown. Within groups 2-4, the profiles are categorized into three subgroups: **(A)** presence of DSA pre-transplant, **(B)** absence of DSA pre-transplant, and **(C)** no transplant.
