# Supplementary material for: Transfusion-induced HLA antibodies are short-lived and rarely recur post-heart transplantation: a single-center retrospective study with implications for virtual crossmatching
Source: Front Immunol. 2026 Jan 21;17:1729124. doi: 10.3389/fimmu.2026.1729124 (PMC12867846; doi:10.3389/fimmu.2026.1729124)

1. No pre-VAD or post-VAD antibodies

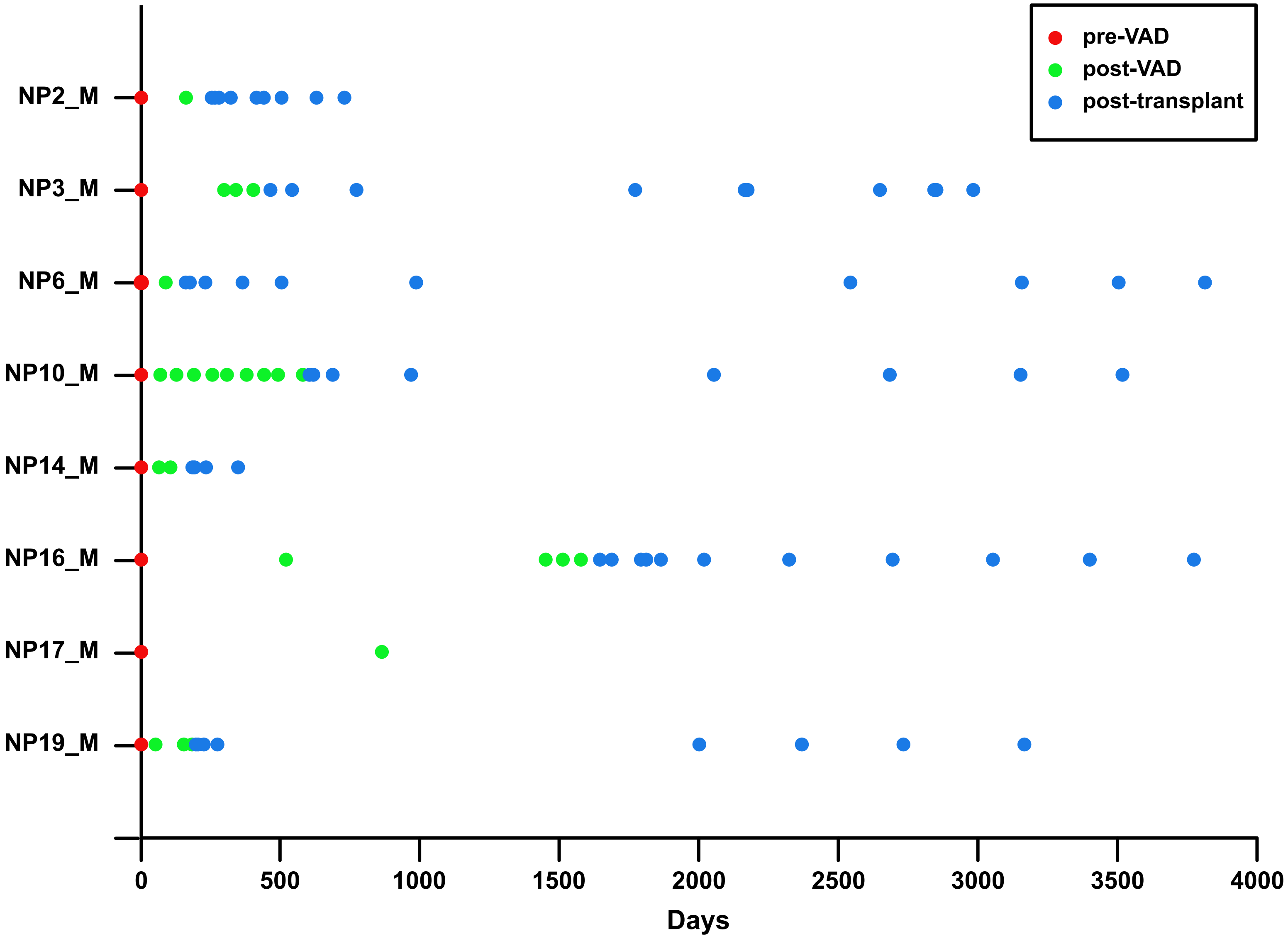

2. No pre-VAD antibodies, with new antibodies formed post-VAD

A) DSA present pre-transplant with no rebound post-transplant

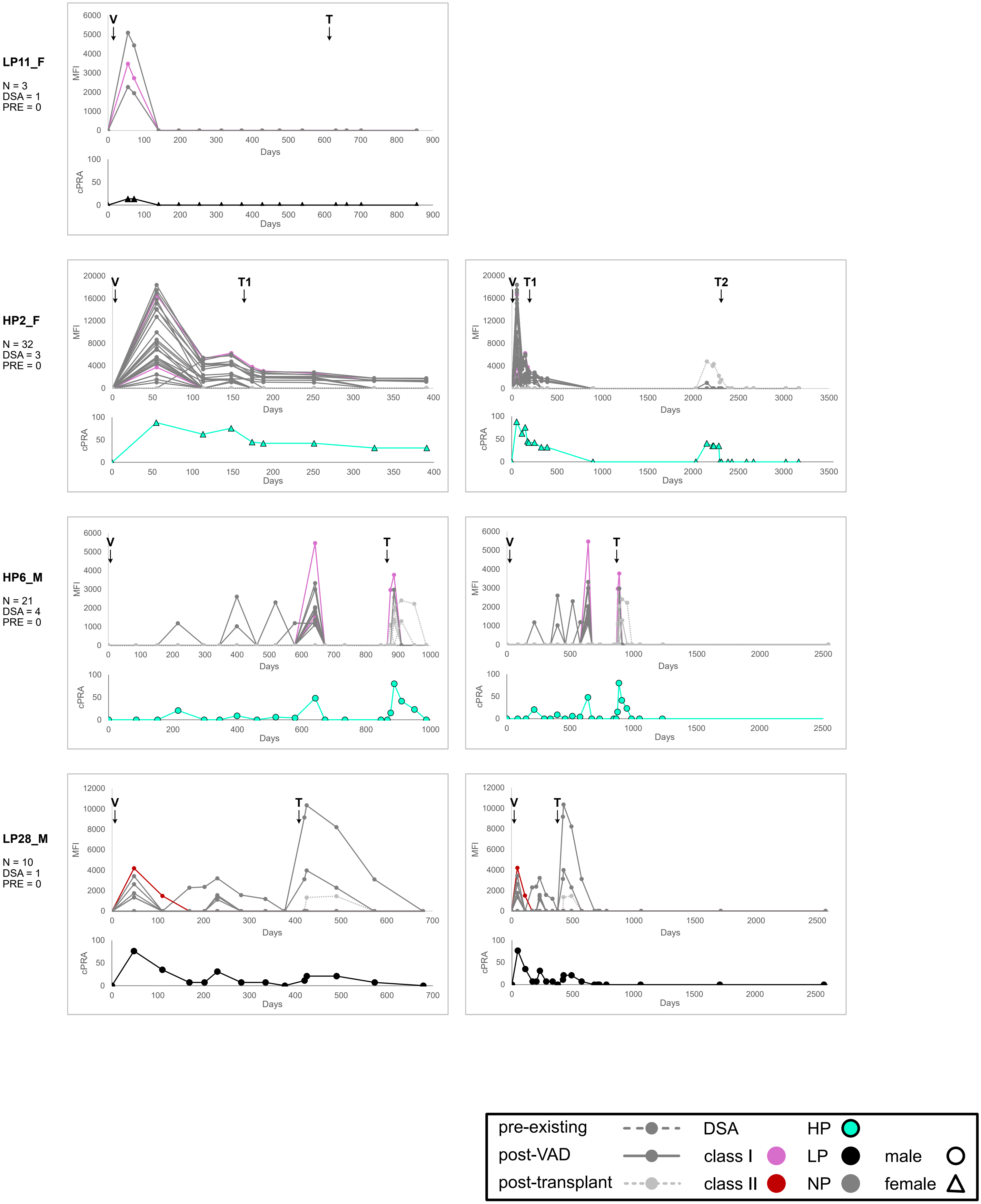

2. No pre-VAD antibodies, with new antibodies formed post-VAD (continued)

B) No DSA present

LP13_M

N = 2
DSA = 1
PRE = 0

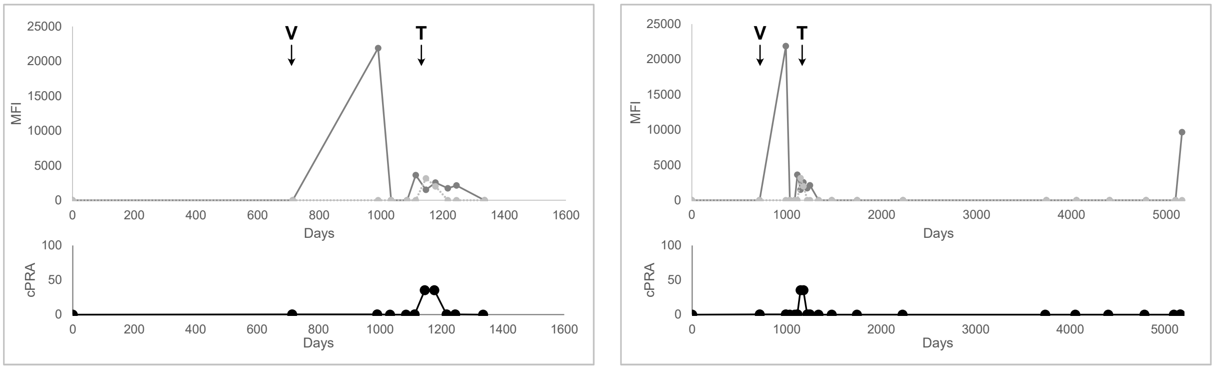

LP3_M

N = 4
DSA =1
PRE = 0

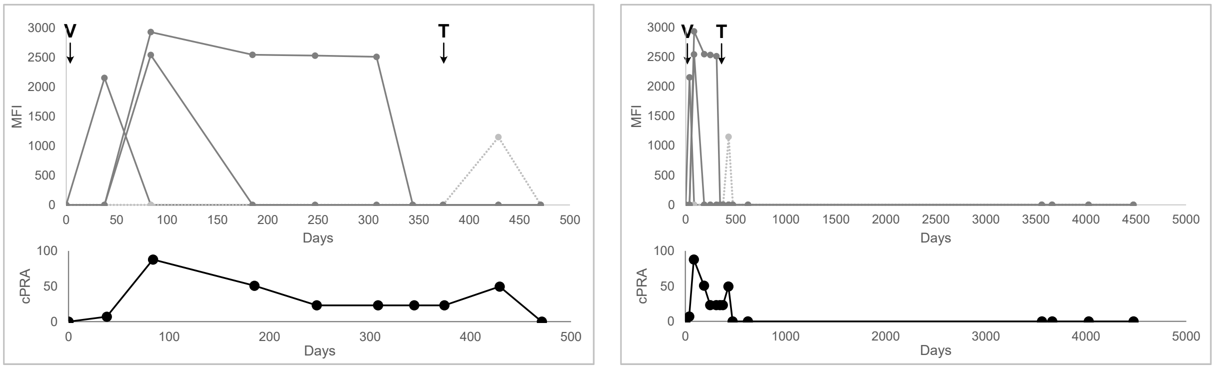

LP7_M

N = 1
DSA = 0
PRE = 0

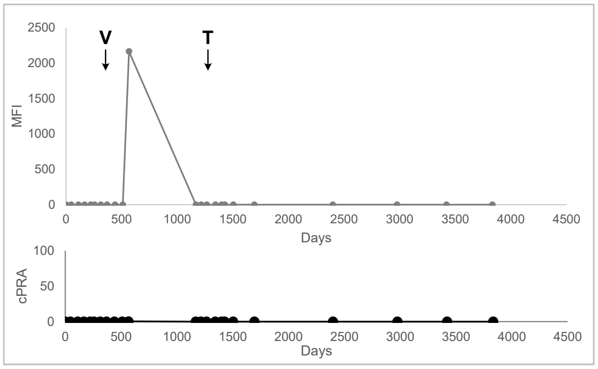

LP16_M

N = 1
DSA = n/a
PRE = 0

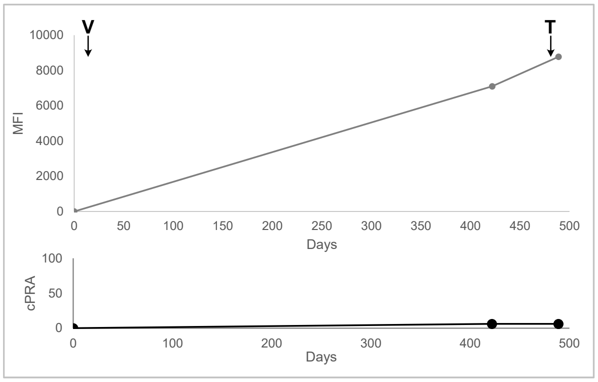

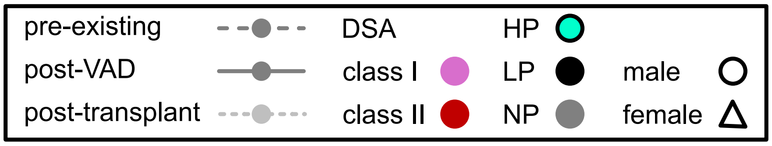

2. No pre-VAD antibodies, with new antibodies formed post-VAD (continued)

B) No DSA present (continued)

LP22_M

N = 3
DSA = 0
PRE = 0

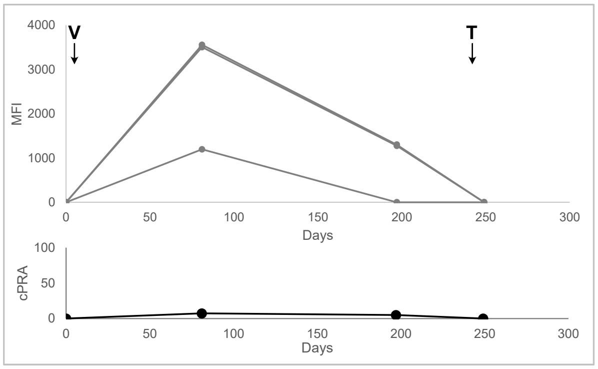

LP19_M

N = 4
DSA = 0
PRE = 0

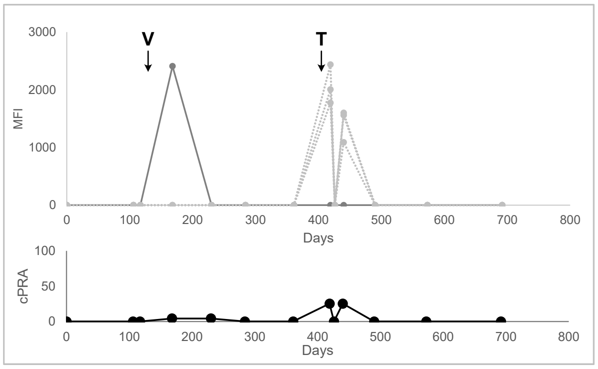

LP23_F

N = 7
DSA = 0
PRE = 0

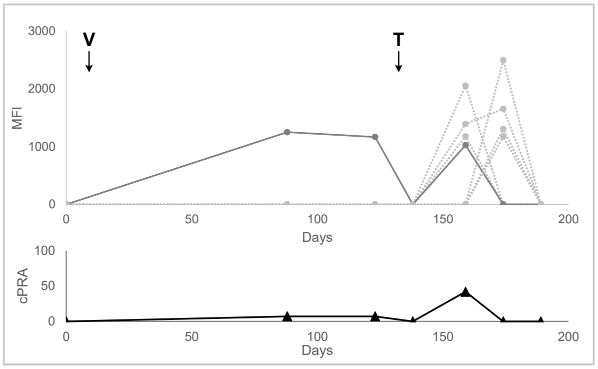

LP26_M

N = 5
DSA = 0
PRE = 0

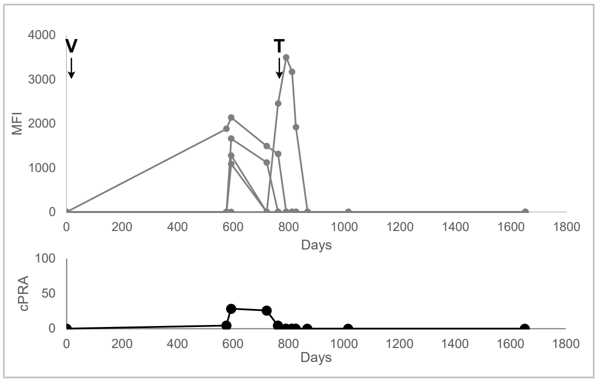

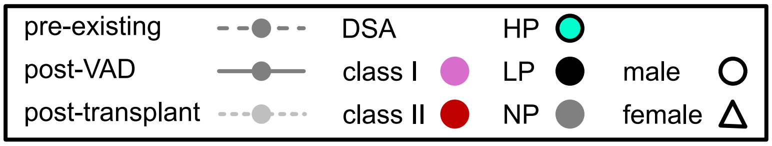

2. No pre-VAD antibodies, with new antibodies formed post-VAD (continued)

B) No DSA present (continued)

LP29_M

N = 8
DSA = 0
PRE = 0

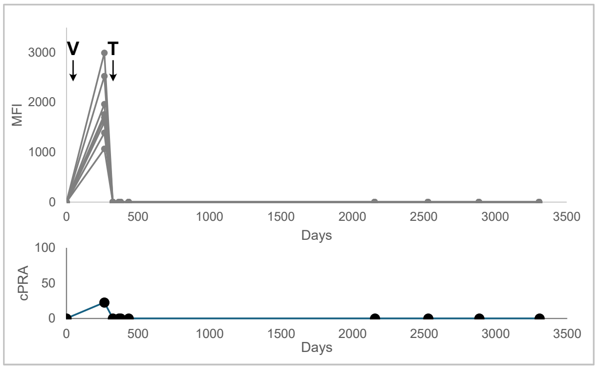

C) No transplant

LP2_M

N = 1
DSA = n/a
PRE = 0

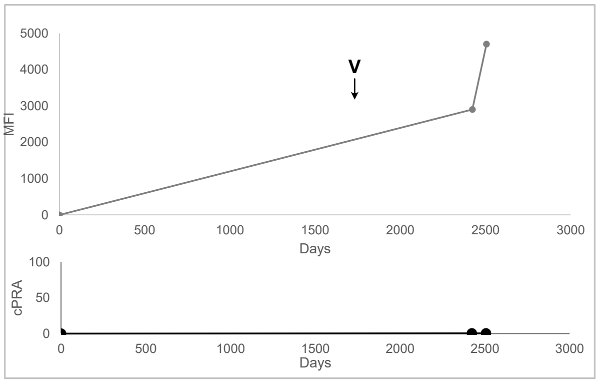

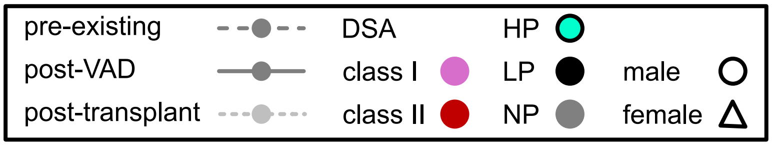

3. Antibodies present pre-VAD, with no new antibodies generated post-VAD

A) DSA present pre-transplant with no rebound post-transplant

NP5_M

N = 4
DSA = 1
PRE = 3

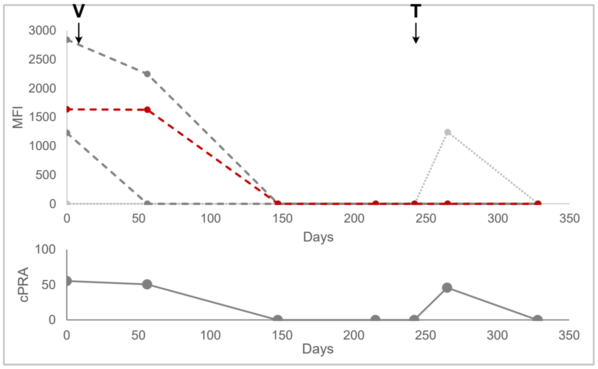

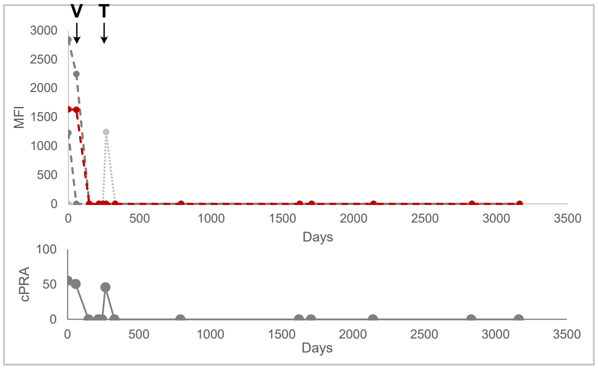

NP12_M

N = 4
DSA = 2
PRE = 4

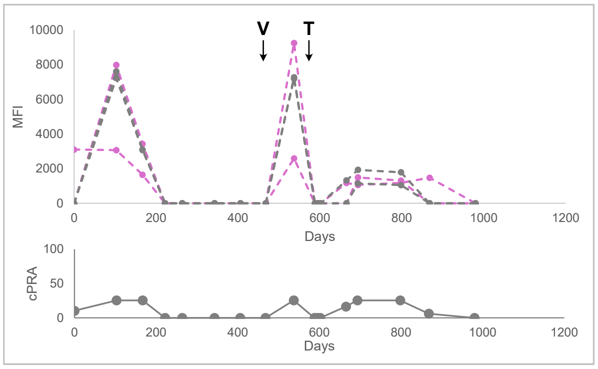

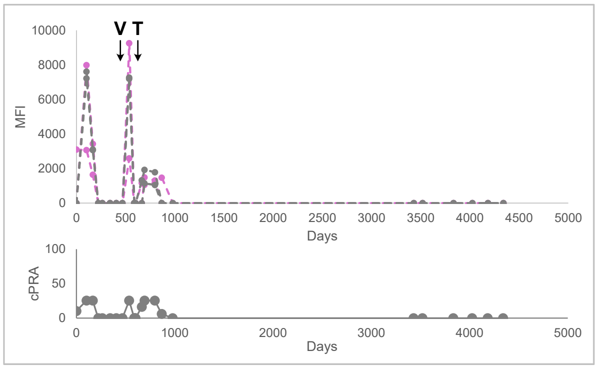

NP21_M

N = 9
DSA = 1
PRE = 8

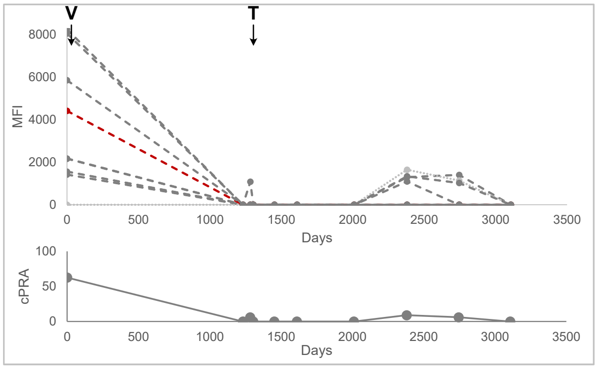

NP20_M

N = 2
DSA = 1
PRE = 2

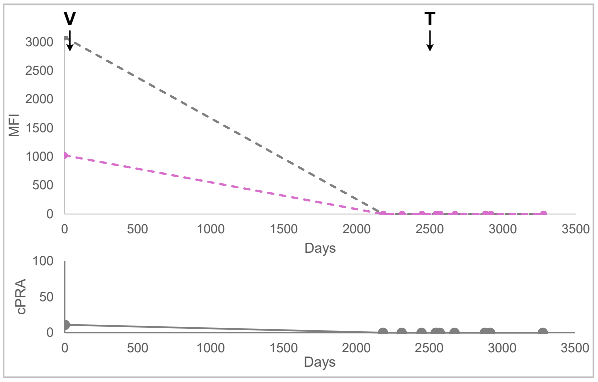

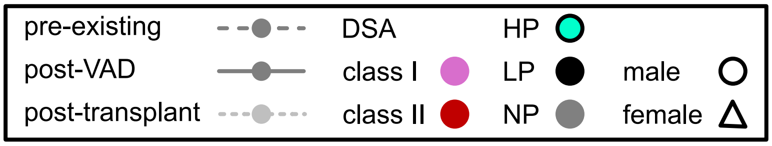

3. Antibodies present pre-VAD, with no new antibodies generated post-VAD (continued)

B) No DSA present

NP15_M

N = 1
DSA = 0
PRE = 1

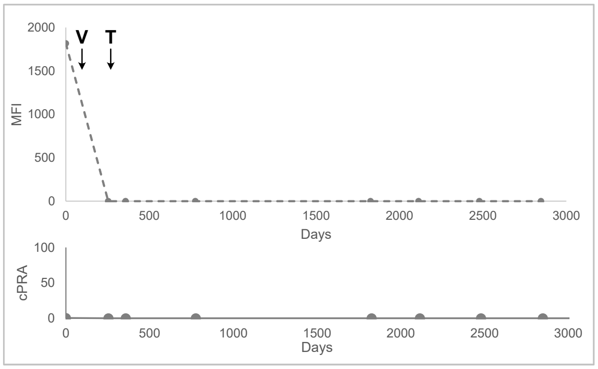

NP13_F

N = 1
DSA = 0
PRE = 1

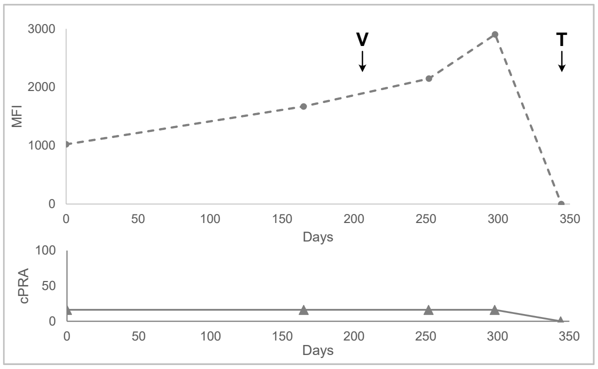

NP18_M

N = 7
DSA = 0
PRE = 7

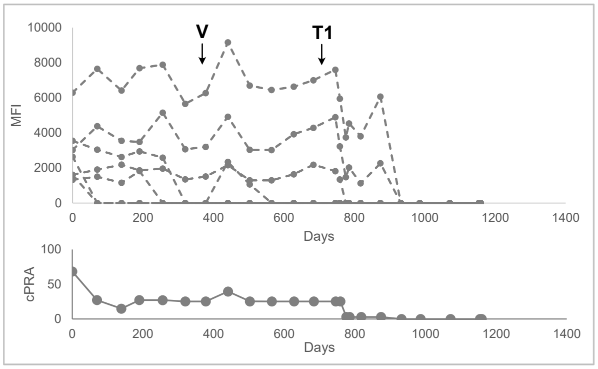

NP9_M

N = 2
DSA = 0
PRE = 2

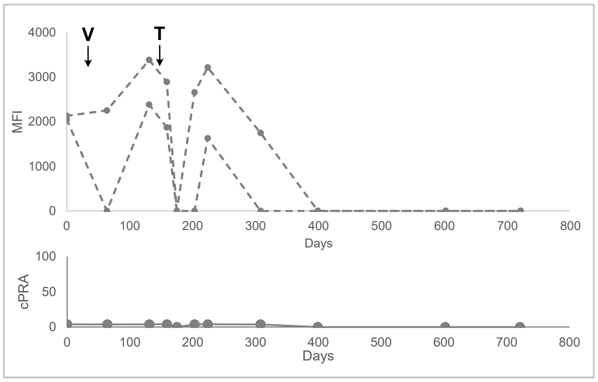

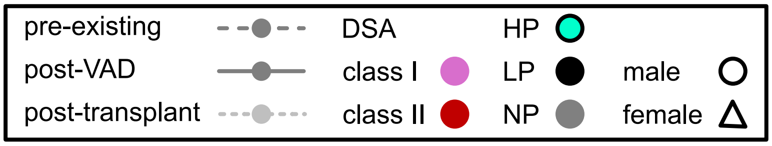

3. Antibodies present pre-VAD, with no new antibodies generated post-VAD (continued)

B) No DSA present (continued)

NP7_F

N = 1
DSA = 0
PRE = 1

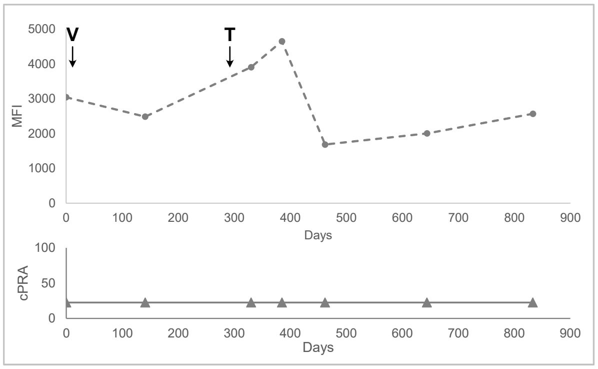

NP11_M

N = 3
DSA = 0
PRE = 1

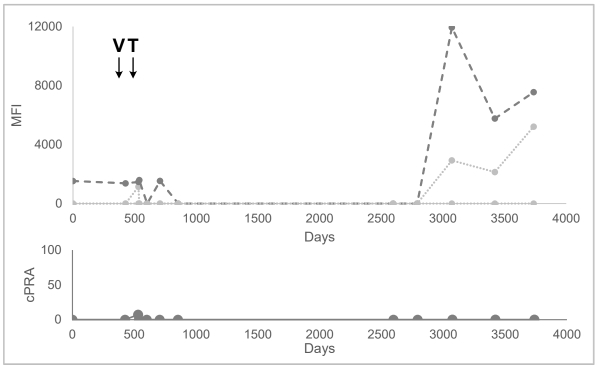

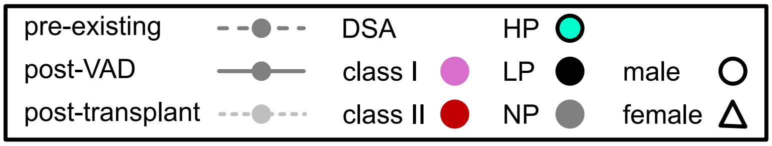

3. Antibodies present pre-VAD, with no new antibodies generated post-VAD (continued)

C) No transplant

NP8_M

N = 5
DSA = n/a
PRE = 5

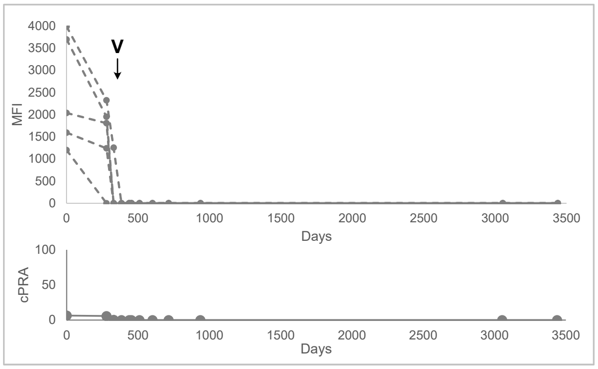

NP1_M

N = 3
DSA = n/a
PRE = 3

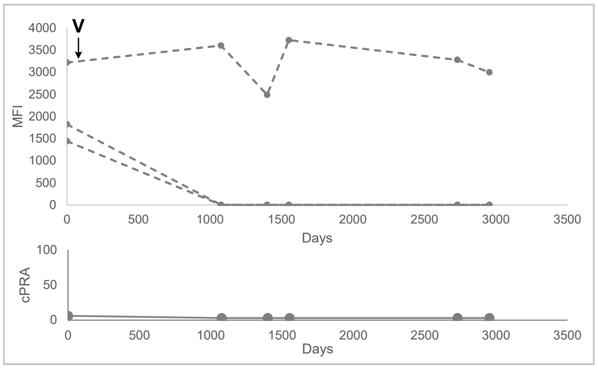

NP4_M

N = 4
DSA = n/a
PRE = 4

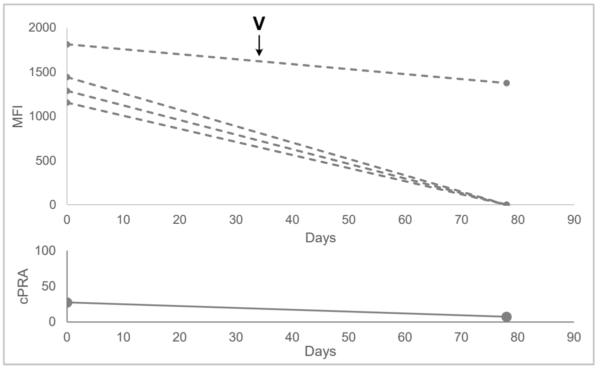

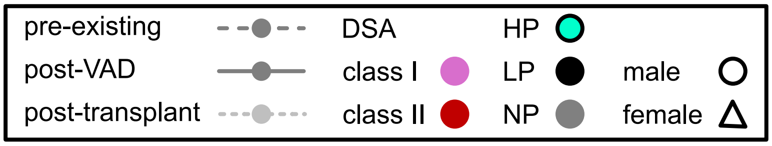

4. Antibodies present pre-VAD, with new antibodies formed post-VAD

A) DSA present pre-transplant with no rebound post-transplant

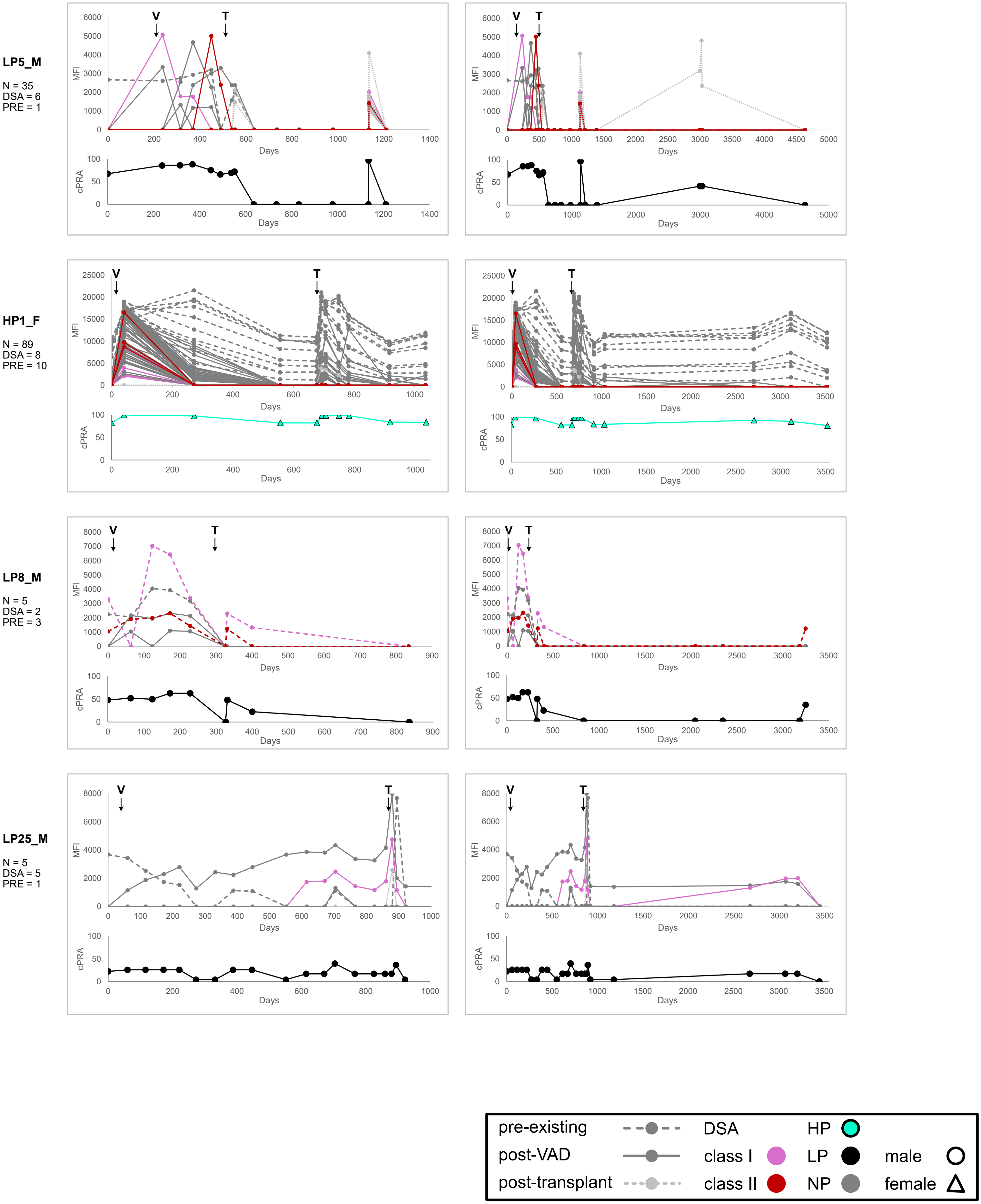

4. Antibodies present pre-VAD, with new antibodies formed post-VAD (continued)

A) DSA present pre-transplant with no rebound post-transplant (continued)

LP21_M

N = 15
DSA = 1
PRE = 3

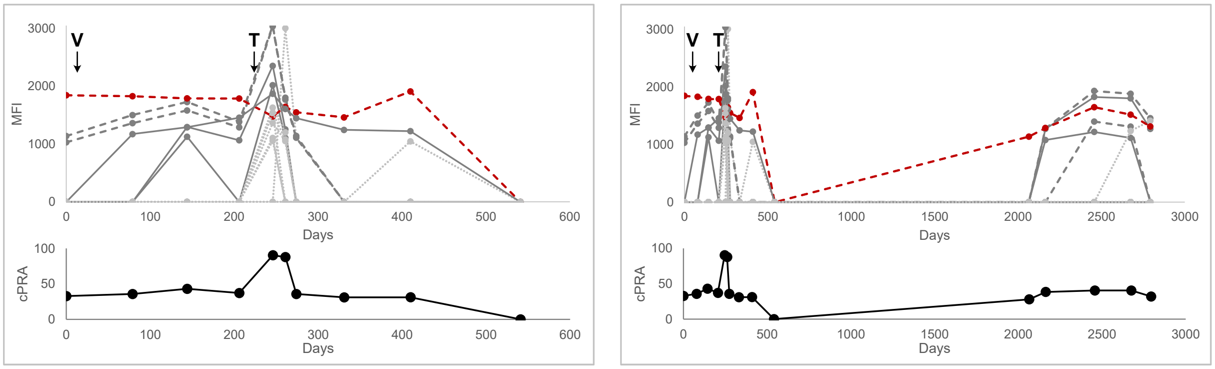

LP30_M

N = 4
DSA = 1
PRE = 2

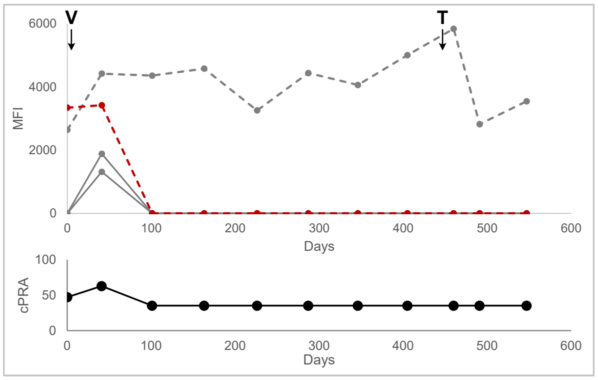

HP5_F

N = 44
DSA =3
PRE = 2

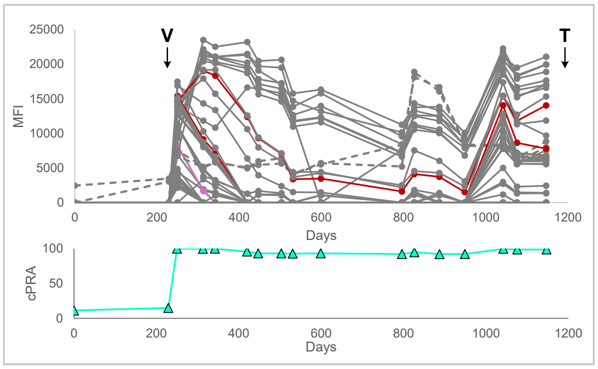

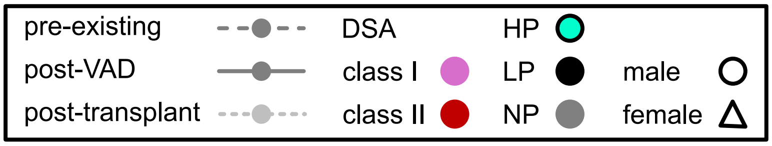

4. Antibodies present pre-VAD, with new antibodies formed post-VAD (continued)

B) No DSA present pre-transplant

LP31_M

N = 4
DSA = 1
PRE = 2

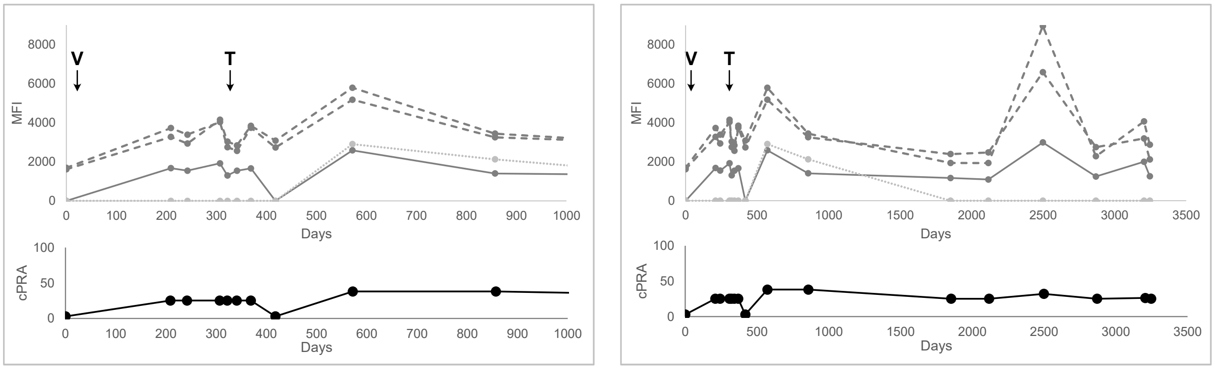

LP4_M

N = 6
DSA = n/a
PRE = 2

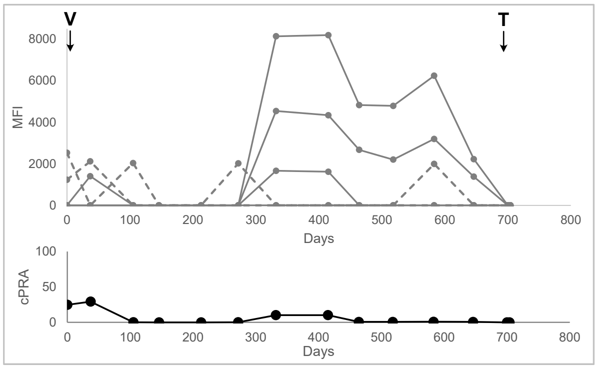

LP9_F

N = 2
DSA = 0
PRE = 1

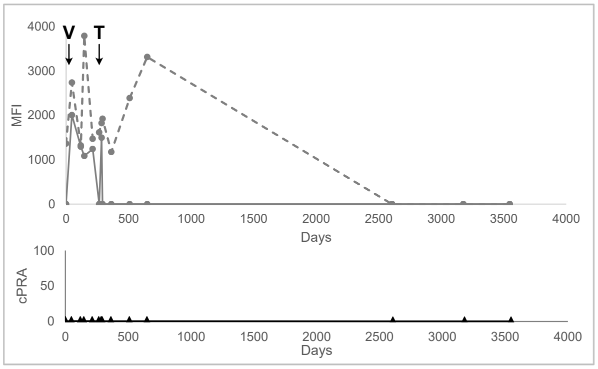

LP10_M

N = 10
DSA = 0
PRE = 5

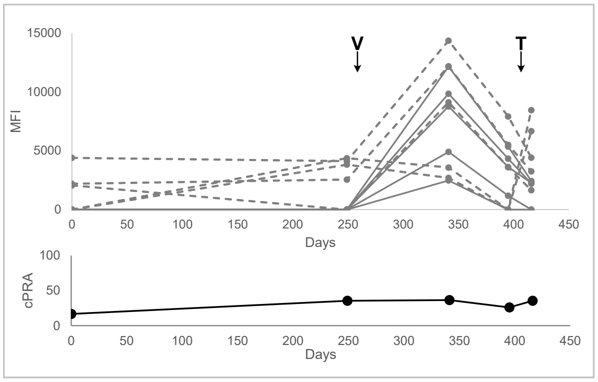

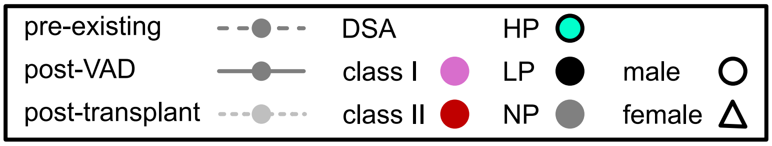

4. Antibodies present pre-VAD, with new antibodies formed post-VAD (continued)

B) No DSA present pre-transplant (continued)

LP12_M

N = 12
DSA = 1
PRE = 2

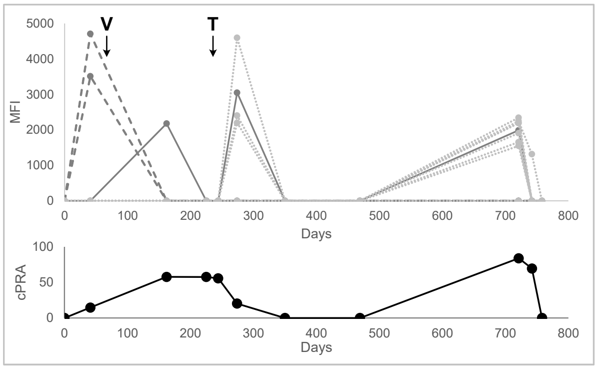

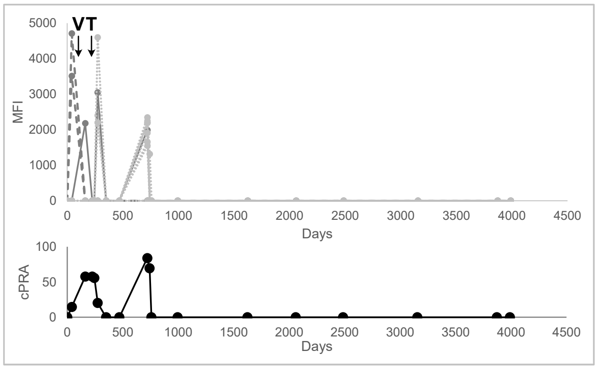

LP14_M

N = 12
DSA = 0
PRE = 6

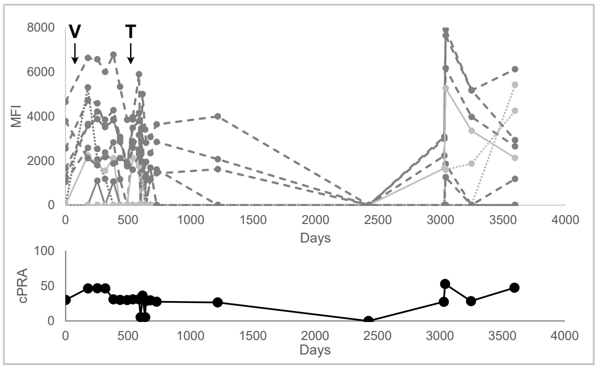

LP15_M
N = 7
DSA = 0
PRE = 5

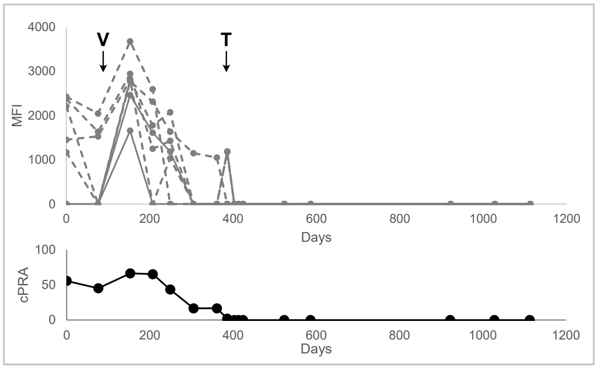

LP17_M

N = 12
DSA = 0
PRE = 4

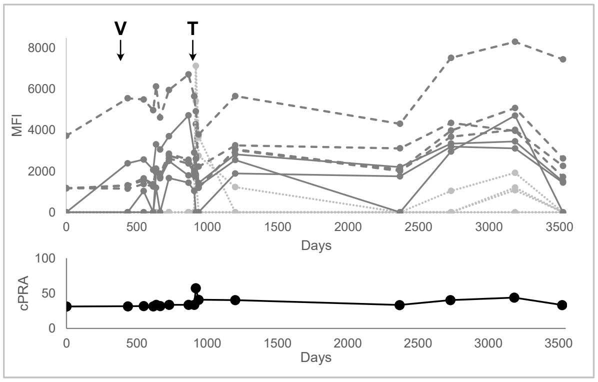

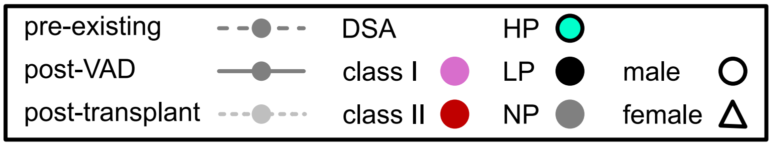

4. Antibodies present pre-VAD, with new antibodies formed post-VAD (continued)

B) No DSA present pre-transplant (continued)

LP24_M

N = 21
DSA = 0
PRE = 6

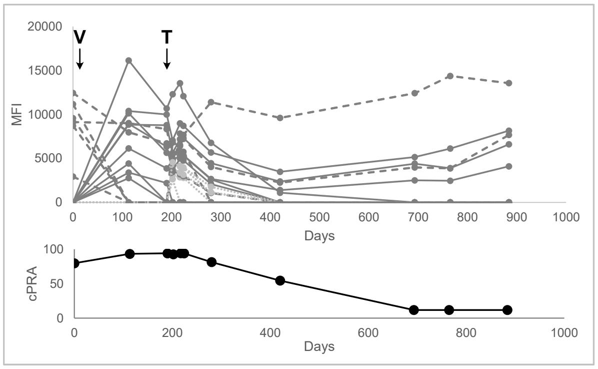

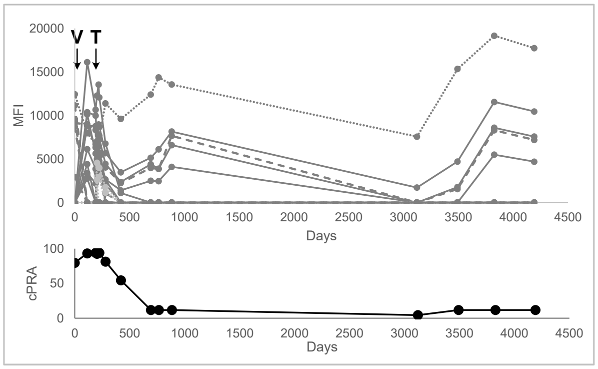

C) No transplant

HP3_M

N = 63
DSA = n/a
PRE = 3

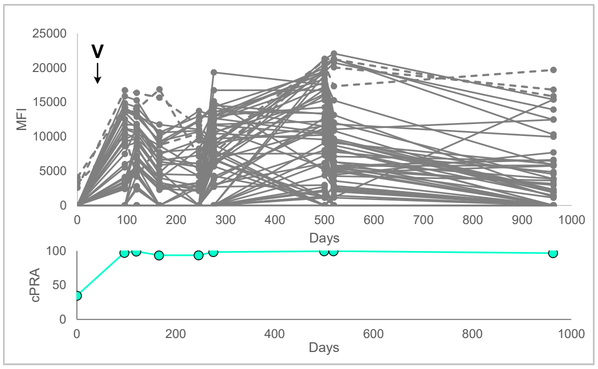

HP4_F

N = 73
DSA = n/a
PRE = 19

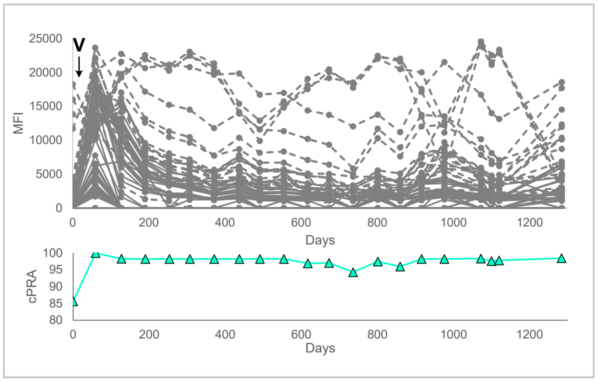

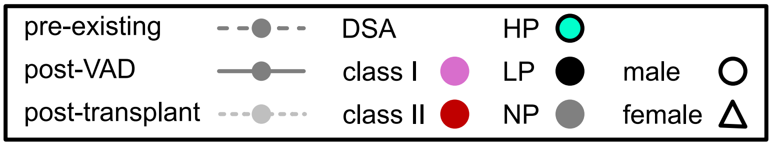

4. Antibodies present pre-VAD, with new antibodies formed post-VAD (continued)

C) No transplant (continued)

HP7_F

N = 100
DSA = n/a
PRE = 62

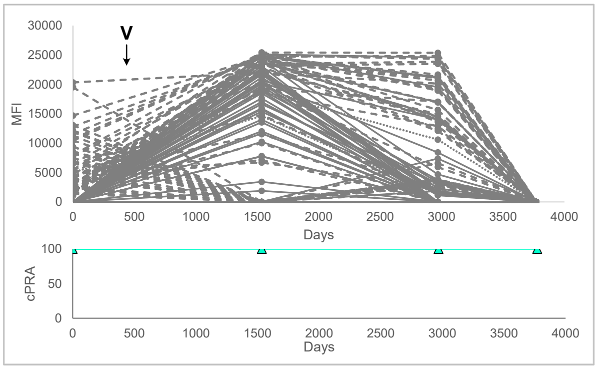

HP8_F

N = 59
DSA = n/a
PRE = 13

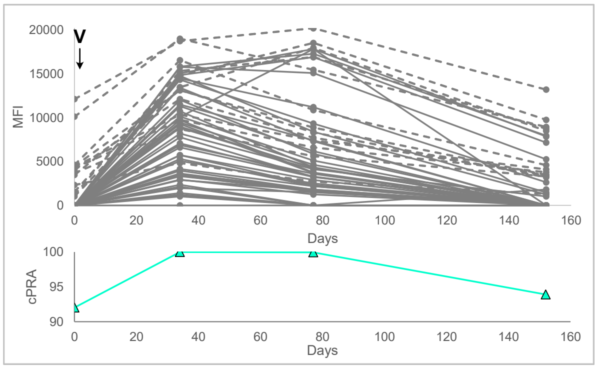

LP1_M

N = 10
DSA = n/a
PRE = 2

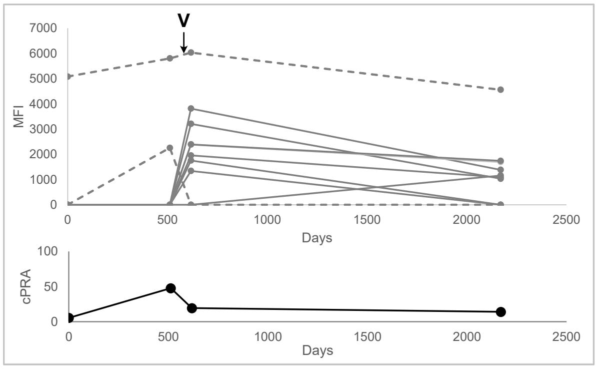

LP18_M

N = 2
DSA = n/a
PRE = 1

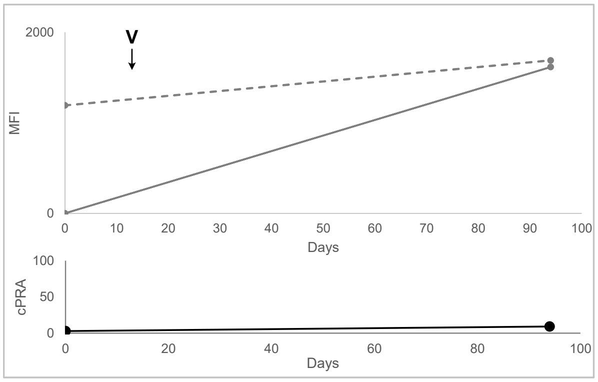

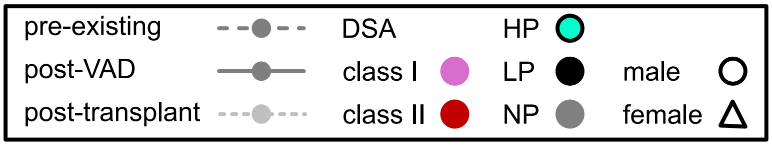

4. Antibodies present pre-VAD, with new antibodies formed post-VAD (continued)

C) No transplant (continued)

LP6_F

N = 2
DSA = n/a
PRE = 1

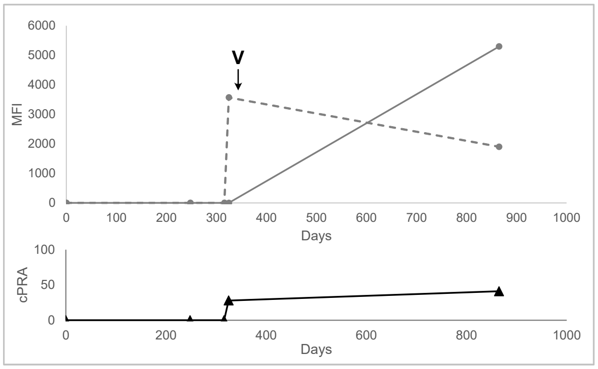

LP27_M

N = 9
DSA = n/a
PRE = 2

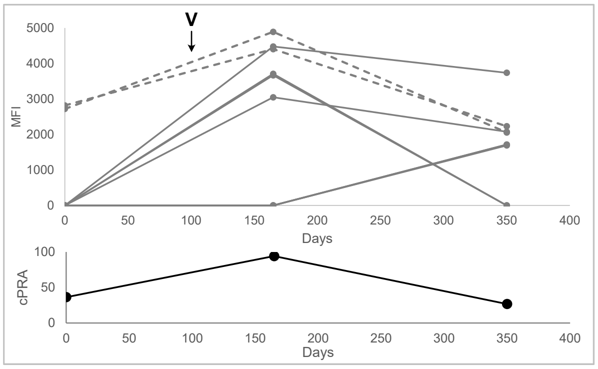

LP20_M

N = 6
DSA = n/a
PRE = 2

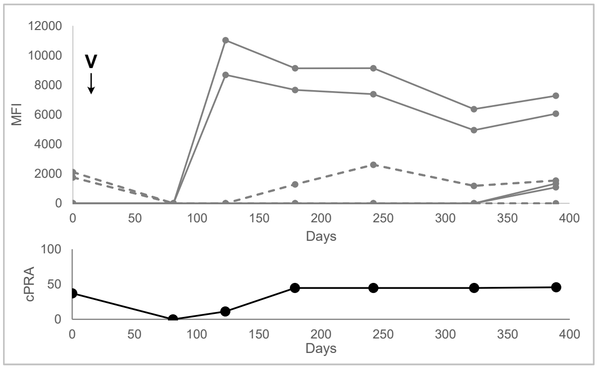

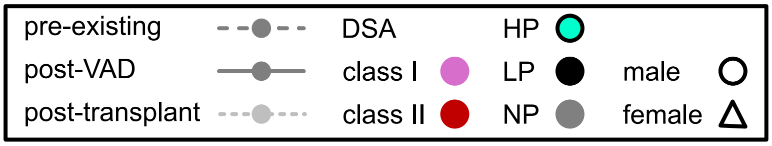

Supplement: Supplementary Figure 1 — Antibody profiles are shown for all cohort members, divided into four major groups outlined in Figure 2. For the group 1 patients who had no preformed antibodies and no new pre-transplant antibodies, the timing of the assays for each patient is shown. Within groups 2-4, the profiles are categorized into three subgroups: (A) presence of DSA pre-transplant, (B) absence of DSA pre-transplant, and (C) no transplant. [file DataSheet1.pdf]
